# Supplementary material for: Neighborhood Physical and Social Environments and Social Inequalities in Health in Older Adolescents and Young Adults: A Scoping Review
Source: Int J Environ Res Public Health. 2023 Apr 11;20(8):5474. doi: 10.3390/ijerph20085474 (PMC10139110; doi:10.3390/ijerph20085474)
Supplement: Supplementary file 1 [file ijerph-20-05474-s001.zip › ijerph-2272621-supplementary/SupplTables_1-3_vf.pdf]

Table S1. Keywords used in the literature search

Population	Outcome	Social environment exposure	Physical environment exposure	Geographic context
Adolescen*<br>**OR**<br>“Young adult*”<br>**OR**<br>“Emerging adult*”<br>**OR**<br>Youth<br>**OR**<br>Teen*	“physical health”<br>**OR**<br>“mental health”<br>**OR**<br>“Wellbeing”<br>**OR**<br>“well-being”<br>**OR**<br>“Social connec*”<br>**OR**<br>“Social participation”	“Social capital”<br>**OR**<br>“Social cohesion”<br>**OR**<br>“Socioeconomic factor*”<br>**OR**<br>“Social condition*”	“built environment”<br>**OR**<br>“physical environment”<br>**OR**<br>Revitalization<br>**OR**<br>Gentrification<br>**OR**<br>“Urban design”<br>**OR**<br>“Public space”<br>**OR**<br>Park<br>**OR**<br>Green*	Neighbo*<br>**OR**<br>Area<br>**OR**<br>“Local environment”<br>**OR**<br>Residen*

Note: The asterisk (*) serves as a truncation symbol to detect variations in a single string of text.

Table S2. Example query string for Scopus database (February 2021)

#	Query string	Results
**1**	TITLE-ABS ("physical health" OR "mental health" OR wellbeing OR well-being OR "social connec*" OR "social participation") PUBYEAR > 1999	369 047 results
**2**	TITLE-ABS (adolescen* OR "young adult*" OR "emerging adult*" OR youth OR teen*) PUBYEAR > 1999	531 625 results
**3**	TITLE-ABS (neighbo* OR area OR "local environment*" OR residen*) PUBYEAR > 1999	4 361 570 results
**4**	TITLE-ABS ("social capital" OR "social cohesion" OR "socioeconomic factor*" OR "social condition*") PUBYEAR > 1999	47 138 results
**5**	TITLE-ABS ("built environment" OR "physical environment" OR revitalization OR gentrification OR "urban design" OR "public space" OR park OR green*) PUBYEAR > 1999	847 565 results
**6**	#1 AND #2 AND #3 AND (#4 OR #5)	228 results

Table S3: Descriptive summary of the 69 articles included in the review

Author	Year	Location	Study design	Age (years)	Health outcome(s)	Environmental exposure(s)	Associations	Setting	Inequalities
**Quantitative studies**									
Soobader et al. [31]	2000	United States	Cross-sectional	0-17	Subjective. Parent-reported measures of children's health and functioning (e.g., presence of a chronic condition, emotional problem, etc.). Behaviour and school problems (behaviour problem index, school suspension and school retention), Parent reported self-perceived child's health status, psychological health: whether the child has ever seen a psychiatrist or counsellor.	Objective. Income distribution measure (county level): Gini index. Measures of poverty: Number of households in county with an income at or less than the Federal poverty level and census tract (proportion of persons whose income is at or below 175% of the Federal poverty line).	County poverty is a predictor of psychological health.	Residential	No
Wickrama et al. [32]	2003	United States	Cross-sectional	13-19	Subjective. Adolescent's depressive symptoms: 19 items from the Center for Epidemiological Studies Depressive Symptoms Scale (e.g., In the past 7 days, was the adolescent "bothered by things that usually don't bother him/her", "felt that he/she could not shake off the blues", "felt depressed, fearful, lonely, and sad").	Subjective: Self-reported collective socialization and formal social integration (parent and adolescents). e.g., parents: "If a neighbor saw your child getting into trouble, would your neighbor tell you about it?", adolescents: "People in this nbh look out for each other". Objective: Concentration of poverty (composite index based on census tracks) Ethnic heterogeneity (census tracts and % of non-whites).	All community exposures were correlated (subjective = inverse association and objective = positive association) with depressive symptoms.	Residential	No
Gutman et al. [33]	2004	Philadelphia, PA, USA	Longitudinal (2 time points at 7-year interval)	12-15 (at wave 1), 19-22 (at wave 2)	Subjective. Mental health (youth depressive symptoms in past 2 months): items assessing how often they felt symptoms such as, hopelessness, loneliness, and unhappiness.	Subjective. Answers provided by primary caregivers and target youth. Nbh cohesiveness (e.g., "do your neighbors have similar views about how to raise children»). Nbh problems (scale, 23 items for Wave 1 and target youth reported on a representative 7-item subset for Wave 2): assault and muggings, drug use, police not caring about problems, and vandalism.	Nbh cohesiveness in adolescence positively associated with fewer depressive symptoms in adolescence (women only) but not in young adulthood (men and women).	Residential	Yes – Subgroup analysis (sex/gender)
Chen et al. [34]	2006	St. Louis, Missouri, USA	Cross-sectional	15-19, 16-19 and 14-18	Subjective. Psychological health: Cognitive Appraisal and Understanding of Social Events (CAUSE) videos, self-reported discrimination questionnaire, self-reported Hostility (Cook-Medley Hostility Scale), threat perception, self-reported Optimism (Life Orientation Test), self-reported self-esteem (Rosenberg's Self-Esteem Scale), self-reported Perceived control (Self-Mastery Scale) Objective. Physiological Health (objective): Blood pressure (BP), heart rate (HR), cortisol (measures stress), body mass index (BMI). .	Objective. SES (neighbourhood level): objective census data from 123 block groups (education, occupation, income, house ownership).	Significant inverse association for SES with BMI, Cortisol, hostility, discrimination, and threat perception. No statistically significant relations for other outcomes (BP, HR optimism, control, and self-esteem).	Residential	No
Day et al. [35]	2007	California, United States	Cross-sectional	N.A. ("adolescents")	Subjective. Adolescent self-reported well-being (how often various feelings of well-being or absence/ability to overcome negative feelings occurred in the past week) and depression (feeling depressed in the last week (yes/no)).	Subjective. Nbh social capital (social cohesion) adolescent's parent or guardian reported. Safety: presence of nbh watch program and any instances of adult respondent's home being broken into in current nbh. Social control: adolescent's response to the "likelihood neighbors would do something about kid's graffiti". Objective. nbh SES (2000 U.S. Census).	Higher social cohesion and higher social capital significantly associated with better mental well-being. Social control is inversely associated with adolescent depression.	Residential	Yes – Effect measure modification (sex/gender and immigrant status)

Meltzer et al. [37]	2007	UK	Cross-sectional	11-16	Objective. Childhood psychopathology: Development and Well-Being Assessment. Interviews by parents and children (if 11 yrs+) rated by clinicians	Subjective: Neighbourhood trustworthiness and safety (e.g., feeling safe walking alone in the neighbourhood) Objective: Neighbourhood-level socioeconomic characteristics (ACORN classification).	Emotional disorder increased when nbh trust and/or safety is lower.	Residential	No
Boyce et al. [38]	2008	Canada	Cross-sectional	14-16	Subjective. Self-rated health: Single item: "Would you say your health is...?" (Excellent, good, fair, poor).	Subjective. Nbh social capital: HSBC items (e.g., "people say 'hello' and often stop to talk to each other in the street", "it is safe for younger children to play outside during the day").	Lower levels of social capital are significantly associated with poor health.	Residential	No
Cicognani et al. [39]	2008	Two small-size deprived towns in North Italy (Marradi (town A) and Brisighella (town B))	Cross-sectional	14-19; 20-27	Subjective. Well Being (Emotional (e.g., 'In the last month, how much of the time did you feel…happy?), Psychological (e.g., 'In the last month, how much of the time did you feel good at managing the responsibilities of your daily life?') and social dimensions): Keyes's (2005) Subjective Well-being instrument (adolescent version).	Subjective. Residential context. Self-reported residential satisfaction: Based on Perceived Residential Environment Quality Scale and its revised Italian version ('People and social relations', 'Social services', 'Recreational services', etc.). Self-reported Sense of community: Sense of Community Scale for Adolescents by Cicognani. Social support: Multidimensional Scale of Perceived Social Support (MSPSS).	When residential satisfaction increases stress is reduced. Demographics and town	Residential	Yes - Subgroup analysis (sex/gender)
Larson et al. [40]	2008	United States	Cross-sectional	0-17	Subjective: Parental assessment of **child's global health status** (good/fair/poor vs. excellent/very good). And **socioemotional problems** (e.g., difficulties with emotions, concentration, behavior, or being able to get along with other people"	Subjective. Parental perception of neighbourhood safety.	Unsafe nbh is positively associated with poorer general health and socioemotional problems.	Residential	No
Maas et al. [42]	2009	Netherlands	Cross-sectional	13-17, 18-25	Objective. Morbidity: derived from routine primary care electronic medical records. 24 disease clusters (7 categories: Cardiovascular, musculoskeletal, mental, respiratory, neurological, digestive, miscellaneous).	Objective. Greenspace: Total percentage of green space in the respondents' living environment (National Land Cover Classification database (LGN4)), urbanicity (measured at the municipal level and derived from Statistics Netherlands).	Prevalence of 15 of the 24 diseases was lower in greener environments (in a 3km radius).	Residential	No
Aslund et al. [44]	2010	Västmanland, Sweden	Cross-sectional	13-18	Subjective. Psychosomatic symptoms (headache, stomachache, feelings of nervousness, feelings of irritation, sleep problems). Musculoskeletal pain. Depressive symptomatology (Depression Self-Rating Scale (DSRS) from DSM-IV).	Subjective. Nbh social capital (e.g., "In my neighbourhood, no-one needs to feel afraid", "It is unsafe to be outside at night in my neighbourhood", "In my neighbourhood, people seldom help each other", "I often see graffiti and damaged objects (park benches, bus stops, streetlights) in my neighbourhood"] answered using a scale from 1 (Strongly agree) to 4 (Strongly disagree). Summation index (7 to 28) separated in 4 quartiles. General social trust: 6 statements (e.g., most people can be trusted).	Nbh social capital and general social trust are inversely associated with psychosomatic symptoms, musculoskeletal pain, and depression.	Residential	No
Borges et al. [43]	2010	Belo Horizonte, Brazil	Cross-sectional	16-17	Subjective. One-item self-rated overall health status ("How would you describe your overall state of health these days, would you say it is very good, good, fair, poor or very poor?").	Subjective. Self-reported social capital: cognitive dimensions (trust in others, perceived helpfulness of neighbors, etc.), behavioural dimensions (participation in community activities, time or money contribution to a community project, group belonging, etc.), bonding vs bridging social capital (youth association with others from	Positive association between bridging SC and health status. Not belonging to a group lowered the odds of reporting poor health. All other associations are not statistically significant.	Residential	No

						different ethnic backgrounds, economic or social status or religious groups.).			
Wu et al. [45]	2010	China (7 cities)	Longitudinal (3 times points at 1-year interval)	11-19	Subjective. Depressive symptoms (time 2): Center for Epidemiological Studies-Depression Scale (e.g., "did you have trouble shaking off sad feelings").	Subjective (time 1). Community social capital (e.g., adolescents: 'Do you feel safe in your nbh?', parents: 'In general, how well do you know your neighbors?')	Higher community social capital is associated with lower depressive symptoms.	Residential	No
DeClercq et al. [46]	2012	Belgium	Cross-sectional	9-18	Subjective. Adolescents' perceived health status and well-being (e.g., "Thinking about last week. Have you felt fit and well? Have you felt full of energy? Have you felt sad?).	Subjective. Community-level social capital (e.g., “People say 'hello' and often stop to talk to each other in the street”, “it is safe for younger children to play outside during the day”). Objective. Average income and "traditional social cohesion scale" aggregated at CT scale (Federal Police, Social Security, the National Institute for Statistics, the Federal Government Department of Statistics and the Roman-Catholic Church databases).	Community social capital is inversely associated with health status and well-being.	Residential	No
Aminzadeh et al. [47]	2013	New Zealand	Cross-sectional	13-17	Subjective. General mood: "In general how have you been feeling?", Life satisfaction: "Are you happy or satisfied with your life?", Wellbeing: WHO-5 Wellbeing Index (means core on scale) (e.g., Whether over the last two weeks "I have felt cheerful and in good spirits", "I have felt calm and relaxed"). The three items/scales were combined into an overall self-reported well-being measure.	Subjective. Neighbourhood social capital (multiple scales): nbh social cohesion, nbh facilities, nbh physical disintegration, membership in community organisations, residential stability. Students' responses aggregated to the neighbourhood level.	Community social capital is inversely associated with well-being.	Residential	Yes – Effect measure modification (cross-level interactions between individual socioeconomic deprivation and neighbourhood measures)
Huynh et al. [48]	2013	Canada	Cross-sectional	11-16	Subjective. Positive emotional well-being: measured using the Cantril ladder. Students were asked to rank their current state of life on a 10-point scale.	Objective (GIS). Public natural space: total area natural space within the buffer: local parks and sport fields, provincial/territorial parks, national parks, other parks, wooded areas, campgrounds, picnic areas, golf courses, driving ranges, national wildlife and migratory areas, botanical gardens, and water bodies, green space (only land features), and blue space (only water features). The percentage of total land within each buffer that consisted of total natural space, green space, and blue space was estimated.	No statistically significant associations.	School	Yes – Effect measure modification (sex/gender and ethnicity)
Ivert et al. [50]	2013	Malmö, Sweden	Cross-sectional	16-17	Subjective. Adolescent mental health: Self-reported version of the Strength and Difficulties Questionnaire (SDQ).	Objective: Nbh SES of small admin units(obj.). Neighbourhood Collective Efficacy (two scales measuring informal social control and social cohesion, subj.). Subjective. Social Disorder (self-reported public drinking, vandalism, drug use in the neighbourhood? etc.)	Social disorder and nbh socioeconomic deprivation are inversely associated with mental health.	Residential	Yes – Subgroup analysis (sex/gender, immigrant background)
Astell-Burt et al. [20]	2014	England, Scotland and Wales (UK)	Longitudinal	15-29	Subjective. Self-reported minor psychiatric morbidity (12-item General Health Questionnaire (GHQ)	Objective. Greenspace: percentage total land cover of green and natural environment (excluding water and private gardens) within residential ward (data circa 2000: vectorization	Greenspace is associated with lower psychiatric morbidity among women across the lifecourse (i.e., from age 15-20 years-old to	Residential	Yes - Subgroup analysis (sex/gender)

						of raster thematic data originating from classified Landsat 5 and Landsat 7 ortho-images).	41 years-old and up. Among men, greenspace is not associated with psychiatric morbidity among 15-20 year-olds, but the inverse association strengthens by age 30 and peaks at ages 41-45.		
Delany-Brumsey et al. [49]	2014	Los Angeles County, CA, USA	Cross-sectional	12-17	Subjective. Mother reported child's internalizing (depressive and anxious) and externalizing (aggressive, hyperactive, and oppositional) behaviour problems: Behavioral Problems Index (28 questions). Mother-reported visit to a psychologist.	Objective. Residential stability of a nbh (% of people in the census track who resided in the same home in 1995 as in 2000), nbh socioeconomic disadvantage (index), Subjective. social capital (aggregated from adult respondent scores at CT scale).	No statistically significant associations.	Residential	No
Marshall et al. [51]	2014	5 cities: Baltimore (Maryland, USA), Shanghai (China), New Delhi (India), Ibadan (Nigeria), and Johannesburg (South Africa).	Cross-sectional	15-19	Subjective. Self-reported general health (5 response options)	Subjective. Nbh social capital: community cohesion (e.g., People in this neighborhood can be trusted, etc.) and degree of connection between individuals (e.g., I feel connected to most people in this neighborhood).	Social capital is positively associated with general health for both males and females in all cities except in Ibadan where there is no statistically significant associations for males.	Residential	Yes – Subgroup analysis (sex/gender)
Assari et al. [53]	2015	City of Flint, Michigan, USA	Longitudinal (3 time points over 8 years)	15 (at wave 1), 21-22 (at wave 5-6), 23-24 (at waves 7-8)	Subjective. Symptoms of depression: Brief Symptom Inventory (e.g., feeling uncomfortable during the past seven days due to symptoms of depression such as feeling hopeless about the future, and having no interest in things).	Subjective. Fear of neighbourhood (e.g., I am afraid of the violence in my neighborhood, I worry that someone in my neighborhood will physically hurt me.)	Increased fear of nbh over time is positively associated with increase in depressive symptoms.	Residential	Yes – Subgroup analysis (sex/gender)
Lee et al. [54]	2015	United States	Longitudinal (2 time points at 1-year interval)	12-20 (at wave 1)	Subjective. Depression: Center for Epidemiologic Studies Depression Scale (CES-D) (e.g., During the last week how often have you felt sad?). Self-esteem (6-item Rosenberg self-esteem scale). Depression was measured at waves 1 and 2.	Subjective: Self-reported nbh collective efficacy (social cohesion and informal social control) and perception of nbh (perceived safety and perceived contentment).<br>Objective: Latino immigrant density and neighbourhood poverty (1990 US Census).	Latino immigrant density is associated with lower depression in immigrant youth but not among non-immigrant Latino youth. Nbh poverty is positively associated with depression. No statistically significant association with depression for nbh safety and collective efficacy.	Residential	Yes – Subgroup analysis (sex/gender and immigrant status)
Novak et al. [56]	2015	Zagreb, Croatia	Cross-sectional	17- 18	Subjective. Self-rated health: "How do you perceive your health?".	Subjective. Nbh social capital (e.g., "Do you feel people trust each other in your neighbourhood (nbh trust)?", "Do you feel that your neighbours step in to criticize someone's deviant behaviour during high school (informal social control)?"	Social capital is positively associated with health.	Residential	Yes – Subgroup analysis (sex/gender)

Novak et al. [57]	2015	Zagreb, Croatia	Cross-sectional	17-18	Subjective. Psychological distress: 6-item Kessler scale.	Subjective. Nbh social capital (e.g., "Do you feel people trust each other in your neighbourhood (nbh trust)?", "Do you feel that your neighbours step in to criticize someone's deviant behaviour during high school (informal social control)?"	Social capital is inversely associated with psychological distress.	Residential	Yes – Effect measure modification (sex/gender)
Saw et al. [58]	2015	Singapore	Cross-sectional	18-25	Subjective. Well-being (positive affect, negative affect, life satisfaction), perceived stress.	Subjective. Greenspace use and access: number of days in a year respondents visited near home and far from home green spaces and typical duration of the visits.<br>Objective: socio-economic status (income per household capita)	No statistically significant associations.	Residential	No
Hogan et al. [59]	2016	Berlin (Germany), Paris (France), London (UK), New York (USA) and Toronto (Canada)	Cross-sectional	25-34	Subjective. Individual happiness (‘How happy are you now?’). Mediators (self-reported health (How is your health in general?) and feeling socially connected to others (I feel connected to the people who live in my neighbourhood)).	Subjective. Residents' ratings of their cities along dimensions of performance (‘It is easy for children in my city to go to a good school’, etc.), and place (‘There are many parks and sports facilities in my city’, etc.).	Place is positively associated with happiness. No association between performance and happiness for young adults.	Residential	No
Novak et al. [55]	2015	Serbia	Cross-sectional	17-19	Subjective. Self-rated health (one-item measure): "How would you estimate your health?" ("very poor", "poor" and "fair" which represented "poor health" and "good" and "excellent" which represented "good health").	Subjective. Nbh social capital (e.g., "Do you feel people trust to each other in your neighborhood?").	No statistically significant associations.	Residential	No
Pabayo et al. [60]	2016	Boston, USA	Cross-sectional	14-19	Subjective. Depressive symptoms: brief adapted version of the Modified Depression Scale. Frequency of five symptoms in the past month: (a) very sad (b) grouchy or irritable, or in a bad mood (c) feel hopeless about the future (d) sleep a lot more or less than usual and (e) have difficulty concentrating on your schoolwork (used as a mediator).	Subjective. Nbh disorder score ((social (i.e., presence or absence of drinking alcohol in public) and physical (i.e., abandoned cars) from the Boston Neighborhood Survey (BNS)), Nbh social cohesion (from BNS also) (e.g., People in my neighborhood can be trusted, People in my nbh are willing to help their neighbors). Nbh danger (data from the Boston Police Department on Counts of criminal homicide, robbery, aggravated assault, burglary, larceny theft, vehicle theft, and arson).<br>Objective. Census Tract (CT) level income inequality (Gini coefficient), Economic deprivation (U.S. Census indicators of proportion of residents living below the poverty level, proportion of households receiving public assistance, and proportion of families with a female head of household).	Lower nbh disorder scores are linked with lower depressive symptoms. Nbh economic deprivation is inversely associated with depressive symptoms. Social cohesion is also associated with depression.	Residential	Yes – Effect measure modification (sex/gender)
Vilhjalmsdottir et al. [62]	2016	Iceland	Cross-sectional	15-16	Subjective. Emotional distress (12 items from a scale designed to measure frequency of symptoms of depression and anxiety). How often during the previous week the statements applied to them (e.g., ‘I was sad or had little interest in doing things', ‘I felt lonely’).	Objective. nbh income inequality measured with the ratio of the mean disposable income of the top 20% highest income households to the mean disposable income of the 20% lowest-income households in the neighborhood. 2006 tax registry and population survey data. Social	When including social capital in the model, income inequality has a positive effect on emotional distress. Social capital has a protective role. An increase	Residential	No

						capital (nbh reciprocity, nbh contentment, sense of security and social trust).	in nbh contentment, sense of security or social trust reduces emotional distress.		
Dzhambov et al. [63]	2017	Plovdiv, Bulgaria	Cross-sectional	15-25	Subjective. General mental health during the past few weeks: 12-item form of the general mental health questionnaire (GHQ-12).	Objective. Road traffic noise.	No statistically significant associations.	Residential	No
Roe et al. [67]	2017	Two areas in Central Scotland	Cross-sectional	16-36	Subjective. Self-reported. Perceived stress (Perceived Stress Scale). Perceived mental well-being (Shorter Warwick-Edinburgh Mental Well-being Scale (SWEMWBS)). Perceived general health, self-reported activity levels, social well-being (e.g., "how strongly do you feel you belong to your neighborhood or local area?").	Subjective. Greenspace: Perceived quality (safety, attractiveness, satisfaction with quality) and distance (. Contact with greenspace (access to garden yes/no and view to green space yes/no). Motivation for visiting green space: motivational drivers for visiting local green space.<br>Objective. Greenspace, i.e., % green space area including public green space, private gardens and other green space such as roadside trees and grass but excluding woodland or forestry areas that were publicly inaccessible.	Greenspace visits, access to a garden and satisfaction with quality of greenspace reduces stress. No view of greenspace from home is linked with higher levels of stress.	Residential	No
Bezold et al. [69]	2018	United States	Cross-sectional	12-18	Subjective. High depressive symptoms: Self-reported - McKnight Risk Factor Survey (MRFS).	Objective. Greenness presence within 250m and 1250m from residence (NDVI). Blue space presence within 250m and 1250m from residence (National Hydrography Dataset and National Centers for Environmental Information dataset	Inverse association between greenness and depressive symptoms. No statistically significant association with blue space.	Residential	No
Dzhambov et al. [64]	2018	Plovdiv, Bulgaria	Cross-sectional	18-35	Subjective. General mental health: the General Health Questionnaire (GHQ-12)	Subjective. Perceived neighborhood greenspace scale (perceived neighborhood greenness, visible greenery from home, accessibility to the nearest structured green space, time spent in green-space, and quality of greenspace Similar measure of perceived blue space.<br>Objective: Greenness: NDVI and tree cover density. Green space: Euclidian distance to nearest structured urban green space. Blue space presence or absence.	Perceived greenspace and greenness are positively associated with mental health.	Residential	Yes – Effect measure modification (sex/gender and ethnicity)
Dzhambov et al. [66]	2018	Plovdiv, Bulgaria	Cross-sectional	18-35	Subjective. Mental health (in last month) measured with a modified 11-item form of the General Health Questionnaire (GHQ).	Objective. Residential noise exposure (L_Aeq). Residential traffic-related air pollution: Nitrogen dioxide (NO_2).	Inverse association between residential noise exposure and mental health. No direct effect of air pollution.	Residential	Yes – Stratified analysis (ethnicity)
Dzhambov et al. [65]	2018	Plovdiv, Bulgaria	Cross-sectional	15-25	Subjective. Mental health (past few weeks): 12-item form of the General Health Questionnaire (GHQ-12)	Subjective. Self-reported measures of availability, access, quality, and usage of greenspace (e.g., Perceived neighborhood greenness: "To what extent is your neighborhood "green?"), Visible greenery from home: "How would you describe the view from your living room (or the room where you spend most of your time awake while at home).<br>Objective. Greenspace: NDVI and SAVI, tree cover density within the 500-m buffer, and Euclidean distance to the nearest urban greenspace	Objective and subjective measures positively and significantly associated with mental health.	Residential	Yes - Effect measure modification & stratified analyses (sex/gender, SES)

Lorenzo-Blanco et al. [76]	2019	Los Angeles, CA and Miami, FL	Longitudinal (2 time points 2 years apart)	13-17 (at wave 1), 15-19 (at wave 3)	Subjective. Adolescent's depressive symptoms: Center for Epidemiologic Studies Depression Scale (CES-D). Hope (Children's Hope Scale). Self-esteem ("I feel that I have a number of good qualities", etc.). Aggressive behaviour ("I am mean to others", etc.). Outcomes assessed at T1 and T3	Subjective: Parents' perceived nbh characteristics (nbh social cohesion and informal social control (Sampson scale), extent of problems (trouble with gangs, graffiti, drugs and violent crime)). Adolescents' perceived nbh characteristics (Questions on how much they felt supported in the nbh, how much they believe the nbh cares about them and the good things young people do).	Higher perceived nbh support associated with higher hope. No statistically significant associations between other nbh exposures and other health outcomes (depressive symptoms, self-esteem, aggressive behaviour)	Residential	No
Colley et al. [71]	2019	Canada (except the territories)	Cross-sectional	12-17, 18-39	Subjective. Self-reported general and mental health (poor, fair, good, very good or excellent). Objective. Obesity (body mass index (BMI) < 25.0kg/squared meter for adults, waist circumference measure) (height and weight).	Objective. Categorical measure of the favourability of the active living environment and Walkability (Can-ALE database).	Inverse association between walkability and outcomes (obesity and health).	Residential	No
Currier et al. [86]	2019	Australia	Cross-sectional	18-25	Subjective. Self-reported Lifetime depression and anxiety: "Has a doctor or other health professional ever told you that you had this condition?" (Depression, PTSD, and "other anxiety disorders"). Self-reported Lifetime suicidal thoughts and behaviors: "Have you ever seriously thought about killing yourself?" and "Have you ever tried to kill yourself?". Self-reported Alcohol and drugs (hazardous) use: Alcohol Use Disorders Identification Test and Australian School Students Alcohol and Drugs Survey.	Objective. Area socioeconomic disadvantage: Australian Bureau of Statistics Socioeconomic Indexes for Areas-Index of Relative Socioeconomic Disadvantage (SEIFA-IRSD).	Greater socioeconomic disadvantage is linked with lifetime depression, lifetime suicidal ideation, and smoking.	Residential	No
Engemann et al. [72]	2019	Denmark	Longitudinal	13-19	Objective. Psychiatric disorders: Danish Psychiatric Central Research Register (contains information on all admissions to Danish psychiatric in-patient facilities since April 1, 1969, and, since 1995, all out-patient visits to psychiatric departments or emergency care). Intellectual disability, Borderline type, Specific personality disorders, anorexia nervosa, eating disorders, obsessive compulsive disorder, neurotic, stress-related, and somatic disorders, single and recurrent depressive disorder, recurrent depressive disorder, bipolar disorder, mood disorders, schizoaffective disorder, schizophrenia, schizophrenia and related disorders, cannabis, substance abuse, any psychiatric disorders. Diagnoses in adolescence (13-19 yrs-old or in adulthood (after 19 yrs-old).	Objective. Greenspace measure: normalized difference vegetation index (NDVI), dataset of yearly individual-level greenspace presence.	Inverse association between NDVI score measured in childhood) and risk of psychiatric disorder later in life. Association stronger for adolescents (13-19 years-old) compared to adults (20 years-old and up).	Residential	No
Estrada-Martinez et al. [73]	2019	United States	Longitudinal (4 times points over 13 years)	11-21 (at wave 1), 24-35 (at wave 4)	Subjective. Depressive symptoms measured at baseline, and each follow up: nine-item version of the Center for Epidemiological Studies Depression Scale (e.g., "You felt sad" and	Subjective: Nbh satisfaction (e.g., "On the whole, how happy are you living in your neighborhood?"). Nbh social cohesion (e.g., "Do people in this neighborhood look out for each other?").	Inverse or positive association between nbh satisfaction and depressive symptoms depending on	Residential	Yes - Effect measure modification and stratified

					"You were bothered by things that usually do not bother you.").	Objective: Nbh SES (income, employment, poverty, etc.). Racial/ethnic and immigrant concentration in nbh. All data collected at wave 1 (baseline).	ethnic group. No association for social cohesion.		models (ethnic background)
Kleszczewska et al. [74]	2019	Poland	Cross-sectional	14- 18	Subjective. Depression: Depression Scale for Children (CES-DC). Stress: Cohen's Perceived Stress Scale (PSS). General satisfaction with life: Cantril's Ladder (one-element visual scale). Self-efficacy: Scale of Perceived Social Self-Efficacy (PSSE).	Subjective: Index of surrounding environment (incl. nbh deprivation (e.g., In the area where you live there is litter, broken glass or rubbish lying around) and nbh social capital (e.g., I could ask for help or a favour from neighbours)).	Inverse associations between environment index and depression and stress. Associations with life satisfaction and self-efficacy are positive.	Residential	Yes – Subgroup analysis (sex/gender)
Laurence [75]	2019	England	Cross-sectional	16-17	Subjective. Well-being: measured with 3 questions on life satisfaction, happiness and feelings life is worthwhile (e.g., 'how satisfied are you with your life nowadays?').	Subjective. Positive and negative local social interactions frequency of a young person's positive social interactions and negative social interactions in their local area. Neighbour trust: (e.g., "most people in my nbh can be trusted"). Objective: socio-economic status and socio-economic resource disadvantage (index).	Inverse association between socio-economic disadvantage and well-being. Positive association between social interactions and well-being.	Residential	No
Malinowska-Cieslik et al. [77]	2019	Poland	Cross-sectional	15-17	Subjective. Positive attitude scale ((PAS) I am proud of my achievements. I always find a reason to be happy. My belief in myself gets me through difficult times in my life. My life has meaning).	Subjective. Nbh social capital (4 statements on social relations in the neighborhood, i.e., social bonds and security)	Positive association between social capital and PAS.	Residential	Yes – Subgroup analysis (sex/gender)
Mavoa et al. [78]	2019	New Zealand	Cross-sectional	12-19	Subjective. Emotional well-being: WHO-5 Well-being Index: (e.g., "in relation to the last two weeks: "I have felt cheerful and in good spirits", "I have felt calm and relaxed"). Depressive symptoms: Reynolds Adolescent Depression Scale short form (e.g., How often do you feel... happy, sad).	Objective. Greenness: NDVI. Water/blue spaces: Euclidean distance from the single mesh block boundary to the coast in meters (< or > 1600m). Presence of inland water within each nbh (water bodies such as creeks may not represented). Biodiversity (diversity of flora species present in the residential nbh): NDVI, total number of different natural land cover types, and presence of native vegetation land cover within the nbh.	Positive association between NDVI and well-being. NDVI, native vegetation nature availability (living in a nbh having 3 or more natural elements) have an inverse relation with depression symptoms. No statistically significant associations for blue space.	Residential	No
Srugo et al. [80]	2019	Ontario, Canada	Cross-sectional	11-20	Subjective. Psychological Distress (The Kessler 6-item Psychological Distress Scale (K6)). Self-Rated Mental Health (scale). Suicide Ideation or Attempt ("In the last 12 months, did you seriously consider attempting suicide?" and "In the last 12 months, did you actually attempt suicide?").	Objective. Greenness based on NDVI	No statistically significant associations.	School	Yes - Effect measure modification (subjective SES and nbg Material and Social Deprivation Index)
Wang et al. [81]	2019	California, USA	Cross-sectional	12-17; 18 and over	Subjective. Psychologial distress: The Kessler 6 (K6) scale.	Objective. Greenness: NDVI	Inverse association between greenness and psychological distress.	Residential	No
Franklin et al. [82]	2020	Southern California, USA	Longitudinal (2 time points 2 years apart)	13-14 (baseline) ;15-16 (follow up)	Subjective. Perceived stress (the 4-item version of the Perceived Stress Scale (PSS), e.g., In the last month, how often have you felt (1) that you were unable to control the important things in your life?).	Objective. Pollution: Annual 2010 and 2012 ambient concentrations of freeway and nonfreeway NRP, Noise: assessed with acoustic models (100m). Greenness: NDVI and enhanced vegetation index (250m). Night light radiance: multispectral polar-orbiting Visible Infrared	Positive associations between pollution and night light radiance, and stress. Inverse association between greenness and stress. No	Residential	Yes – Effect modification (household income)

						Imaging Radiometer Suite (VIIRS) satellite provide views of artificial light at night (ALAN).	statistically significant association for noise.		
Oluwaseyi et al. [83]	2020	South Africa	Longitudinal	15-24	Subjective. Incident depression (as a proxy for mental health): Centre for Epidemiologic Studies Depression Scale (CES-D).	Subjective. Nbh social capital (measured with neighbourhood trust and generalized trust: Are neighbours and strangers (2 different items) likely to return their wallet if they lost it in the neighbourhood and the wallet contained their identification document in addition to some money). Measurement of crime and safety levels in nbh (low vs high).	Positive relation between crime and incident depression. Inverse association between social capital and depression.	Residential	No
Poulain et al. [84]	2020	Leipzig, Germany	Cross-sectional	10-19	Subjective. Emotional problems: self- or parent-report version of the Strengths and Difficulties Questionnaire (SDQ). The "emotional problems" scale of the SDQ contains questions on the emotional wellbeing of children and adolescents	Objective. Greenspace (%, sum of agriculture, lawn, bushes/young trees and trees) and streets (sum of railways and streets) within 50, 100 and 400 m of the home.	Positive relation between % of streets and emotional problems.	Residential	Yes - Effect measure modification (family SES and sex/gender)
Shen [95]	2020	United States	Cross-sectional	6-17	Subjective. Parent-reported child's mental health (index). Based on MD diagnosis of anxiety problem, depression, behavioural or conduct problem or any other mental health condition.	Objective. Nbh built environment (presence or absence of specific structural infrastructures and facilities (e.g., sidewalks/or walking paths, parks/playgrounds).	Presence of recreation center and library associated with lower risk of mental health disorders.no association between parks/walking paths and mental health.	Residential	Yes - Subgroup analysis (ethnicity)
Hirota et al. [90]	2021	United States	Cross-sectional	13-18	Subjective. Mental disorders (mood, anxiety, behaviour, substance use, and eating) assessed during CIDI interview.	Subjective. Cognitive social capital measured with parent-reported nbh trust and reciprocity (how neighbours help and look out for each other). Composite international diagnostic interview	Inverse association between SC and mental disorders.	Residential	No
Oswald et al. [87]	2021	Australia	Cross-sectional	18-24	Subjective. Mental well-being (14-item self-report Mental Health Continuum-Short Form (MHC-SF)). Mental illness (self-report Kessler Psychological Distress Scale (K-10)). Complete Mental Health State based on their MHC-SF and K-10 scores (Flourishing, Languishing, Struggling, or Floundering in life).	Subjective. Perceived neighbourhood built environment and naturalness, Greenspace and/or blue space within walking distance from home.	Having a greenspace/blue space within a walking distance reduced the risk of languishing. Nbh naturalness reduced the risk of languishing and floundering. Highly built environment increased the risk of floundering,	Residential	No
Zhang et al. [88]	2021	China	Cross-sectional	All ages (87% 20-25)	Objective. Emotional response (from photo) Positive response index assesses the net presentation of positive emotion, that is evaluated as happy score minus sad score.	Objective. Greenspace and blues pace cover and height (for greenspace) from satellite imagery	Positive association between green spaces and happy scores. Negative relationship between blue space and sad scores.	24 parks across 10 cities	Yes – Effect measure modification (sex/gender)
Barnhart et al. [68]	2022	United states	Cross-sectional	12-17	Subjective. Adolescent flourishing i.e., “feeling good and functioning effectively” (three items: interest in and curiosity in learning new things, ability to complete the tasks they start, and ability to remain calm when challenged)	Subjective. Neighbourhood social cohesion (four items: perceptions of neighbours helping one another, watching out for children, safety of children, and knowing where to go for help). Physical Neighbourhood environment (four items: presence of walkways, parks, recreation centers, and libraries)	Positive association between neighbourhood cohesiveness and higher levels of flourishing ($b = 0.111$, $p<0.01$). Significant relation between physical environment and higher levels of flourishing ($b = 0.043$, $p<0.05$)	Residential	No

Bloemsa et al. [91]	2022	Netherlands	Cross-sectional	11-20	Subjective. Mental well-being assessed with the five-item Mental Health Inventory (MHI-5)	Objective. Greenspace (NDVI and percentage of urban agricultural and natural green space in circular buffers (300m, 1000m, 3000m) around home. Air pollution: annual average concentrations (PM10 and PM2.5), nitrogen dioxide, PM2.5 absorbance and oxidative potential. Road traffic and railroad noise (Daily average at adolescents' homes)	No association between traffic noise and poor mental well-being. Single-exposure models: Higher NDVI associated with lower odds of poor mental well-being for all buffers (e.g., 300m: OR = 0.88, 95%CI [0.78-0.99]). Model accounting for type of green: Association with agricultural and natural greenspace in 3000m buffer, but not for urban greenspace. Higher exposure to ambient air pollution associated with higher odds of poor mental wellbeing (e.g., OR 1.23 [95% CI 1.08–1.40] per 0.28 nmol DTT/min/m3 increase in OP^{DTT})	Residential	Yes – Effect measure modification (sex/gender, parental education)
Carrillo-Alvarez et al. [92]	2022	Spain (Barcelona)	Cross-sectional	14-18	Subjective. Psychological distress (Kessler k-6 scale)	Subjective. Neighbourhood social capital (two items: "Do you feel people trust each other in your neighbourhood (neighbourhood trust)?"; "Do you feel that your neighbours step in to criticize deviant behavior among high school students (informal social control)?".	Inverse association between social capital and psychological distress. Neighbourhood trust (r = -0.204, p<0.001), informal social control (r = -0.113, p<0.01)	Residential	No
Hunduma et al. [93]	2022	Ethiopia (Harari regional state)	Cross-sectional	13-19	Subjective. Mental health problems (strength and difficulty questionnaire (SDQ-25))	Subjective. Social capital (12-item social capital questionnaire for adolescent students (SCQ-AS)). Two neighbourhood-related domains: neighbourhood social cohesion and neighbourhood trust	High community social cohesion reduces mental health problems (OR = 0.66 95%CI [0.55-0.89]). Higher neighbourhood trust associated with lower mental health problems (OR = 0.50, 95%CI [0.42-0.60])	Residential	No
Larson et al. [41]	2022	United States	Cross-sectional	18-24 (80%)	Subjective. Emotional distress: participants were asked the extent to which they were currently experiencing each of five emotions (afraid, irritable, sad, preoccupied, and stressed).	Objective. Urban setting (population density of >300 people per square kilometer), Nearby park area (Km^2, county-level: national/state parks and local parks). Park data: Esri. Vegetative cover: NDVI at the ZIP code level.	Inverse relation between area of national/state parks and emotional distress. No other exposure presented significant associations.	Residential	No
Sadler et al. [94]	2022	United States (Baltimore, MD)	Cross-sectional	16-23	Subjective. Internalizing symptoms composite: Anxiety (BAI 21-item questionnaire) and depressive symptoms (CES-D)	Subjective. Neighbourhood social cohesion (3-item scale, e.g. "People in my neighborhood are willing to help each other.").<br><br>Objective. Inequitable housing practices (redlined, blockbusted or gentrified areas)	Gentrification: no significant direct or indirect (via nbh cohesion) effect on internalizing symptoms. Moderated mediation effect for girls (IE = 0.06, 95% bootstrapped CI = 0.01 to 0.14). Blockbusting: Moderated mediation effect	Residential	Yes – Effect measure modification (sex/gender)

						for boys only, lower social cohesion in blockbusted area which result in higher rates of internalizing symptoms. (IE = 0.04, 95% bootstrapped CI = 0.01 to 0.08). Redlining: No significant effect.		
Stahlmann et al. [96]	2022	Germany	Cross-sectional	0-17	Subjective. Mental health (Strength and Difficulties Questionnaire (SDQ))	Subjective. Social infrastructure (SI) places (KiGGS) presence and distance to (playgrounds, sports fields, parks, swimming pools). Assessed with the questions: “Are there play or sports facilities in your current location that are within easy reach of your child?”, “How long does it take [your child] to reach the following places/facilities by foot?” For each item (playground, sports field/hall, indoor playground, swimming pool/hall, park/public greenspace, forest, beach/lake/stream/river (in the following blue spaces), and bus or train stop).	Inverse relation (with gradient) between number of SI places and risk of having total difficulties and peer problems. (SI=0: OR = 1.59, 95%CI [1.19-2.11) and SI = 3: OR = 1.14, 95%CI [0.99-1.33]). Higher odds of emotional symptoms and hyperactivity/inattention when access to less than four SI places.	Residential	Yes – Stratified analyses (parental SES)
Zewdie et al. [98]	2022	Ethiopia (Addis Ababa)	Cross-sectional	18-26	Subjective. Psychological health (continuous scores on two measures: Difficulties (Strengths and Difficulties Questionnaire (SDQ)) and Depressive symptoms (Patient Health Questionnaire 8-item scale (PHQ-8)).	Objective. Greenspace (NDVI) in administrative units (sub-cities) and subunits (kebeles).	Greenspace is significantly associated with difficulties (b = -1.89, p = 0.02) but not with depressive symptoms.	Residential	Yes – Effect measure modification (sex/gender, income)
Zhang et al. [89]	2022	New Zealand (Auckland)	Cross-sectional	10-19 (95% 13-18)	Subjective. Emotional well-being (Health Organization-5 Well-being Index (WHO-5)). Depressive symptoms (Reynolds Adolescent Depression Scale-short form (RADS-SF)).	Objective. Greenspace (NDVI) (neighbourhood percentage and distance to edge of nearest urban greenspace) Euclidean buffers of 100m, 300m, 800m and 1600m around meshblock addresses.	Unadjusted models: 100m: Negative association between NDVI and well-being (b = -0.05, p<0.05). 1600m: Positive relation between NDVI and well-being (b = 0.081, p<0.001) and negative relation between percentage and well-being (b = -0.051, p<0.05) Adjusted model: No significant association between outcomes and exposures.	Residential	No
**Qualitative studies**								
Mmari et al. [52]	2014	Baltimore (USA), Shanghai (China), Johannesburg (South Africa), Ibadan (Nigeria)	Cross-sectional	15-19	Subjective. Perceived health.	Subjective. Physical and social environment.	Physical environment as a factor negatively influencing health, especially the presence of litter, the lack of recreation spaces for youth, and the presence of vacant buildings, were mentioned.	Residential	Yes – Subgroup analysis (sex/gender)

		New Delhi (India).							
Rigg et al. [79]	2019	Chicago, IL, USA	Cross-sectional	10-18	Subjective. Well-being.	Subjective. Community violence, lack of safe parks, scarcity of organized recreational activities.	Neighborhood violence, lack of safe parks and scarcity of organized recreational activities have negative impacts on well-being.	Residential	No
Cole et al. [70]	2019	Eastern USA	Cross-sectional	18-30	Subjective. Resilience and mental health	Subjective. Community member influences, building/land environment, diversity.	Parks, rivers, sport courts, quietness have a positive impact on mental health while litter, noise, lack of cleanliness, etc. have a negative impact.<br><br>Presence of positive and negative community members influences.<br><br>Diversity has a positive impact on resilience and mental health.	Residential	Yes – Subgroup analysis (sex/gender and ethnicity)
Benninger et al. [85]	2021	Cleveland, OH, USA	Cross-sectional	13-17	Subjective. Health and well-being.	Subjective. Crime and safety, built environment, social influence, and community activities.	Crime has a negative impact on health and well-being (less physical activity, higher stress and anxiety).<br><br>Built environment such as libraries and community centers, and community activities, especially sports are considered to have a positive impact on adolescents' health.<br><br>Youth social influences have both a positive and a negative impact on the outcomes.	Residential	No
Thompson et al. [97]	2022	UK	Cross-sectional	14-18	Subjective. Health and well-being.	Subjective. Local environment (greenspace, park accessibility, pollution, noise, etc.).	Local environment has both a positive and a negative impact on youth health and well-being.	Residential	No
**Mixed-methods studies**									
Glendinning et al. [36]	2007	Siberia, Novosibirsk region, Russia	Cross-sectional	14-15	Subjective. Mental health (self-worth and depression) and self-rated general health: measures of general self-esteem (10 items), depressive mood (6 items), psychological distress (12 items) and subjective health complaint (iritability, anxiety, difficulties	Subjective. Prospects for youth (e.g. 'The future looks good for young people who stay on here'), quality of life (e.g. 'This is a good place for young people like me to stay'), feelings of support (e.g. 'There are people here I can rely on to talk to [outside of my family]'), Community	Community constraints have an inverse effect on self-worth and a positive correlation with depression in small communities.	Residential	Yes – Subgroup analysis (level of urbanicity)

					sleeping, headaches and abdominal pain (8 items)). Measure of self-rated health generally ('How do you assess the state of your health in the course of the previous year?').	constraints (feelings of constraint (e.g. 'It's hard to be yourself here'), feelings of insecurity (e.g. 'It's not safe to go out at night here') and lack of amenities (e.g. 'There's nothing for young people to do here')). City vs small communities.	Poor future prospects and community constraints are positively correlated with depression. Associations between nbh exposures and general and mental health are not clearly presented.		
Teixeira [59]	2016	Homewood neighborhood of Pittsburgh, PA, USA	Cross-sectional	14-19	Subjective. Well-being, hopelessness, and vulnerability.	Objective. Rate of housing abandonment in nbh. Subjective. Housing abandonment perception.	Abandoned/vacant housing have a negative impact on individual and community well-being.	Residential	No
